# Epigenetics in Ocular Diseases

**DOI:** 10.2174/1389202911314030002

**Published:** 2013-05

**Authors:** Melissa M Liu, Chi-Chao Chan, Jingsheng Tuo

**Affiliations:** 1 Laboratory of Immunology, National Eye Institute, National Institutes of Health, Bethesda, MD; 2 Johns Hopkins University School of Medicine, Baltimore, MD, USA

**Keywords:** Age-related macular generation, Cataract, Diabetic retinopathy, Epigenetics, DNA methylation, microRNA.

## Abstract

Epigenetics pertains to heritable alterations in gene expression that do not involve modification of the underlying genomic DNA sequence. Historically, the study of epigenetic mechanisms has focused on DNA methylation and histone modifications, but the concept of epigenetics has been more recently extended to include microRNAs as well. Epigenetic patterning is modified by environmental exposures and may be a mechanistic link between environmental risk factors and the development of disease. Epigenetic dysregulation has been associated with a variety of human diseases, including cancer, neurological disorders, and autoimmune diseases. In this review, we consider the role of epigenetics in common ocular diseases, with a particular focus on DNA methylation and microRNAs. DNA methylation is a critical regulator of gene expression in the eye and is necessary for the proper development and postmitotic survival of retinal neurons. Aberrant methylation patterns have been associated with age-related macular degeneration, susceptibility to oxidative stress, cataract, pterygium, and retinoblastoma. Changes in histone modifications have also been observed in experimental models of diabetic retinopathy and glaucoma. The expression levels of specific microRNAs have also been found to be altered in the context of ocular inflammation, retinal degeneration, pathological angiogenesis, diabetic retinopathy, and ocular neoplasms. Although the complete spectrum of epigenetic modifications remains to be more fully explored, it is clear that epigenetic dysregulation is an important contributor to common ocular diseases and may be a relevant therapeutic target.

## INTRODUCTION

Epigenetics pertains to the study of genomic structural modifications that affect gene expression but do not alter the underlying DNA sequence. Epigenetic mechanisms for the regulation of gene expression include DNA methylation, histone modifications, and microRNAs. Cytosine may be methylated at the C5 position, commonly in the context of CpG dinucleotides, and methylation of constitutive heterochromatin regions and promoter regions is generally associated with decreased gene transcription. Aberrant methylation in CpG islands is associated with genomic instability and has been recognized as an early genomic alteration in certain human tumors [1]. Histones, particularly at their N-terminal tails, are subject to a variety of covalent modifications, including methylation, acetylation, sumoylation, and phospho rylation. Numerous enzymes are involved in chromatin modification, the best studied of which are histone deacetylases (HDACs), histone acetyltransferases (HATs), and DNA methyltransferases (DNMTs). These modifications regulate gene transcription by affecting the accessibility of DNA and the recruitment of DNA binding proteins [2]. MicroRNAs are small RNAs that mediate post-transcriptional downregulation of gene expression [3]. Environmental exposures and diet alter epigenetic patterning, [4] and there is an age-related decrease in global levels of DNA methylation [5]. As a result, epigenetic modifications may be particularly relevant in the pathogenesis of complex age-related diseases with known environmental risk factors and modulators [4]. The role of epigenetic modification has been established in a variety of human diseases, including cancer, imprinting disorders, neurological disorders, and autoimmune diseases [6, 7]. In this review, we focus on the role of epigenetics in common ocular diseases.

## DNA METHYLATION

Epigenetic mechanisms are critical regulators of gene expression in ocular development and homeostasis. DNA methyltransferases 1, 3a, and 3b (*Dnmt1*, *Dnmt3a*, and *Dnmt3b*) are robustly expressed in early embryonic stages, and differential nuclear localization patterns in rod and cone photoreceptors suggest that DNA methylation may contribute to cell fate determination in retinal neurons [8]. In mice, retina specific deletion of DNA methyltransferase 1 (*Dnmt1*) disrupts retinal differentiation and leads to rapid retinal degeneration, with effects that are particularly pronounced in photoreceptors [9]. These results demonstrate that DNA methylation is necessary for the development and survival of retinal neurons. DNA methylation has also been shown to maintain normal cell type-specific gene expression patterns in photoreceptors and in non-photoreceptor retinal cells [10].

### Age-Related Macular Degeneration

Age-related macular degeneration (AMD) is the leading cause of irreversible central vision loss in the elderly, and it is estimated to affect more than 1.75 million individuals in the United States [11]. The pathogenesis of AMD involves a complex interaction of both genetic and environmental risk factors [12-15]. The role of epigenetics in AMD, however, is incompletely understood. A DNA methylation study using peripheral blood mononuclear cells was performed in one pair of monozygotic twins and two pairs of dizygotic twins with discordant AMD phenotypes to identify differentially methylated regions across the genome [16]. In the twins with AMD, there were 231 genes that showed altered methylation patterns in their promoter regions. Notably, the promoter region of *IL17RC*, the receptor for IL-17A and IL-17F, was hypomethylated in the twins with AMD. AMD patients also had increased frequency of IL-17RC positive monocytes in the peripheral blood and increased macular expression of IL17-RC transcripts and protein relative to non-AMD controls. Taken together, these results suggest that AMD patients may be more susceptible to Th17 mediated inflammatory responses and that this type of inflammation may contribute to AMD pathogenesis. In a recent study, bisulfite sequencing was used to perform genome-wide methylation profiling in post-mortem retinal pigment epithelium (RPE) and choroid from patients with AMD. Hypermethylation of the promoter regions of two glutathione S transferase isoforms (GSTM1 and GSTM5) was identified, which correlated with decreased mRNA and protein levels [17]. Glutathione S transferases are an important in the defense against reactive oxygen species, and epigenetic downregulation of these enzymes may mediate increased susceptibility to oxidative stress in AMD. 

### Cataract, Pterygium, and Retinoblastoma

DNA methylation profiling was performed in human lens epithelial cells for αA-crystallin (*CRYAA*), which encodes a structural protein in the lens, to evaluate the role of epigenetic regulation at this locus in the development of age-related cataract. CRYAA transcript and protein levels are both downregulated, and the CpG island in the *CRYAA* promoter is hypermethylated in human age related cataract [18]. In pterygium, a benign condition in which wedge-shaped epithelial fibrovascular proliferation of the conjunctiva extends onto the cornea, altered methylation patterns were detected at CpG loci near the genes encoding transglutaminase 2 (*TGM2*), metalloproteinase 2 (*MMP2*), and *CD24*. Hypermethylation at the promoter region of *TGM2* led to decreased transcript and protein levels, and hypomethylation of intergenic regions of *MMP2* and the promoter region of *CD24 *led to increased transcript and protein levels. As these genes play a role in extracellular matrix remodeling and cell adhesion, their dysregulation may contribute to pterygium development [19]. In retinoblastoma, hypermethylation has been observed in the promoter regions of *MSH6*, *CD44*, *PAX5*, *GATA5*, *TP53*, *VHL*, *GSTP1*, *MGMT*, *RB1*, and *CDKN2* genes that participate in a variety of cancer-related pathways, including DNA repair, tumor suppression, and cell-cell interactions [20]. Evidence also suggests that the microenvironment of the anterior chamber of the eye may induce epigenetically mediated downregulation of tumor cell expression of the chemokine receptor CXCR4, which is associated with invasion and metastasis [21]. The studies of DNA methylation in ocular diseases are summarized in (Table **1**).

## HISTONE MODIFICATIONS

A study was performed in the streptozotocin-induced diabetic rat model to evaluate epigenetic regulation of manganese superoxide dismutase (*Sod2*) and its potential role in the development of diabetic retinopathy [22]. Hyperglycemia increased interactions between the promoter region and the NF-κB transcription factor p65. It also increased the level of histone H4 lysine 20 trimethyl (H4K20me3) and histone H3 lysine 9 acetyl (H3K9ac) marks at the *Sod2 *promoter and enhancer regions. H4K20me3 is a repressor of gene expression whereas H3K9ac is an activator, and their coexistence suggests that hyperglycemia affects the epigenetic regulation of *sod2* in a complex manner. Here, H4K20me3 has a dominant effect, and *sod2 *gene expression is downregulated. This may increase susceptibility to oxidative stress and promote progression of diabetic retinopathy. A brief summary of this study is included in (Table **1**). Silencing of normal gene expression has been noted as an early change associated with retinal ganglion cell (RGC) death in experimental models of glaucoma and other optic nerve diseases, and an evaluation of apoptotic RGCs in mice showed that histone deacetylase 3 (HDAC3) and histone H4 deacetylation may be critical mediators of gene silencing [23].

## MICRORNAs

MicroRNAs (miRNAs) comprise another mechanism of epigenetic regulation and are single-stranded non-coding RNA molecules that contribute to post-transcriptional downregulation of gene expression. They bind complementary regions of target mRNAs, most commonly at the 3’ untranslated region (3’-UTR), and decrease translation or mark them for degradation by the RNA-induced silencing complex (RISC). miRNA genes are transcribed in the nucleus, and several functionally related miRNAs may be expressed in a single primary miRNA transcript (pri-miRNA). The pri-miRNA is cleaved by Drosha into precursor miRNAs (pre-miRNAs) with characteristic hairpin structures, which are subsequently exported from the nucleus. The pre-miRNAs are processed again by Dicer in the cytoplasm to yield mature 19-25 nt miRNAs [24]. It has been estimated that approximately 30% of human genes are regulated by miRNAs [25]. Although some miRNAs function as binary off-switches in the repression of target proteins, many miRNAs are attenuated regulators that fine-tune mRNA and protein expression levels for up to hundreds of targets in a cell type-specific manner [3, 26].

miRNAs play an important role in development, ocular homeostasis, and disease, [27] and miRNA expression profiling has been performed in various ocular tissues [28]. In the human limbal-peripheral corneal epithelium, miR-143/145 are highly expressed and have been identified as key regulators of corneal epithelium formation and integrity [29]. The miR-29 family, particularly miR-29a, also regulates the synthesis of extracellular matrix in the human trabecular meshwork [27]. miR-124a levels are carefully regulated in development, and elevated levels lead to anomalies in retina and optic nerve development [30]. Microarray based miRNA profiling has been performed in the developing mouse retina and has revealed several temporally differentially expressed miRNAs [31].

### Dicer and Retinal miRNAs

Retina-specific knockdown of Dicer, and the subsequent decrease in all retinal mature miRNAs, led to a decrease in scotopic and photopic electroretinogram (ERG) responses and diffuse progressive retinal degeneration [32]. Photoreceptor rosettes, which are pathological structures in retinoblastoma, diabetic retinopathy, and retinitis pigmentosa, also formed as a result of miRNA depletion. Dicer deletion in the embryonic retinas of mice resulted in increased differentiation of ganglion cells and horizontal cells, decreased late progenitor cells, and consequently decreased rod photoreceptor and Muller glia differentiation [33]. These results demonstrate that Dicer and miRNAs are indeed critical for normal retinal development, structure, and function. In the RPE of patients with geographic atrophy AMD, an advanced subtype of the disease, the level of DICER1 was found to be decreased, resulting in the accumulation of *Alu* element transcripts and RPE toxicity [34]. In mice, RPE-specific Dicer1deletion also led to widespread RPE cell degeneration. In addition to its critical role in miRNA processing, DICER1 appears to also be required for an entirely independent activity, related to repetitive element transcript degradation and RPE cell survival.

The specific roles of individual miRNAs remain to be more thoroughly investigated. A challenge inherent in the study of individual miRNAs is that multiple miRNAs often act on a single target, and a single miRNA often acts on multiple targets. Cyclin-dependent kinase inhibitor 1A (p21Cip1/Waf1), for example, is directly targeted by 28 miRNAs [35]. As a result, the knockout of a single miRNA may not always lead to an observable phenotype. In the case of miR-182, for example, which is highly expressed in the mouse eye, knockout did not lead to any detectable deficit [36]. Nevertheless, numerous studies have been performed to evaluate the expression and function of specific miRNAs in the retina [37]. A microarray based analysis of miRNAs differentially expressed in adult mouse retina, brain, and heart showed that at least 78 miRNAs are expressed in the retina and that 21 may be retina specific. A sensory organ-specific miRNA cluster comprised of miR-96, miR-182, and miR-183, expressed in photoreceptors and bipolar cells, was also identified [38]. miR-96, miR-182, and miR-183 are downregulated and miR-1, miR-133, and miR-142 are upregulated consistently in multiple mouse models of retinitis pigmentosa, suggesting that this may be a miRNA signature of retinal degeneration [39, 40]. In the lens epithelium, the miRNA let-7b has also been associated with increased risk for the development of age-related cataract [41].

miR-204 is highly expressed in the RPE, neural retina, lens, and ciliary body, and it plays a critical role in the proper development of the optic cup and lens by regulating *Meis2* and modulating *Pax6* activity. In the human trabecular meshwork, miR-204 has been found to target genes in multiple functional pathways, including apoptosis, endoplasmic reticulum stress, and inflammation [42]. In the medaka fish, knockdown of miR-204 led to microphthalmia, coloboma, and abnormalities in the lens [43]. In the RPE, miR-204 and the closely related miR-211, are the two most highly expressed miRNAs. They maintain epithelial integrity and directly target TGF-βR2 and the tight junction repressor SNAIL2. In a human fetal RPE model, miR-204 knockdown led to decreased transepithelial resistance and decreased expression of claudins 10, 16, and 19 [44]. miR-204/211 has also been shown to be a critical factor in maintaining RPE differentiation [45]. Taken together, miR-204/211 may be a relevant target for diseases involving loss of RPE integrity and RPE dedifferentiation. 

### Ocular Inflammation and Pathological Angiogenesis, and Uveal Melanoma

miRNAs expression levels are also altered in the context of ocular inflammation. In the murine experimental autoimmune uveoretinitis model, miR-142-5p and miR-21 were found to be upregulated, and miR-182 was found to be downregulated [46]. In cultured human RPE, there is an increase in immunoregulatory miR-155 upon exposure to pro-inflammatory cytokines that is mediated by JAK/STAT signalling [47]. Further evaluation of the activity of this miRNA may also be relevant to better understand the role of the RPE in ocular inflammatory diseases, such as uveitis. 

miRNAs are significant regulators of developmental and pathological angiogenesis [48]. Pathological angiogenesis is a hallmark of a variety of ocular neovascular diseases, including exudative/neovascular AMD, diabetic retinopathy, and retinopathy of prematurity. Endothelial cell specific Dicer deletion in mice leads to defects in post-natal angiogenesis in response to a variety of pro-angiogenic signals, including vascular endothelial growth factor (VEGF), malignancies, limb ischemia, and wound healing [49]. These changes were mediated in part by the Dicer deletion induced increase in thrombospondin-1, an anti-angiogenic agent. 

miR-126, highly enriched in endothelial cells, is critical for endothelial cell migration and blood vessel integrity. In mice, miR-126 deletion led to aberrant angiogenesis during development and partial embryonic lethality [50]. In zebrafish, morpholino mediated miR-126 knockdown led to reduced diameter, disrupted blood vessel organization, and compromised blood vessel integrity [51]. miR-126 functions to promote VEGF activity by directly repressing *SPRED1 *and *PIK3R2*. Recently, p21-activated kinase 1 (*pak1*) was also identified as a target of miR-126 and a mediator of blood vessel integrity [52]. The miR-23/27/24 clusters have also been implicated in regulating angiogenesis and choroidal neovascularization (CNV). These miRNAs are enriched in endothelial cells. Inhibition of miR-23/27 effectively suppresses the development of retinal vasculature and is protective against laser-induced CNV in mice [53]. miR-23/27 directly target Sprouty2 and Sema6A, negative regulators of angiogenesis. In an ischemia-induced model of retinal neovascularization, seven miRNAs (miR-106a, miR-146, miR-181, miR-199a, miR-214, miR-424, and miR-451) were upregulated, and three miRNAs (miR-31, miR-150, and miR-184) were downregulated. Interestingly, intraocular injection of pre-miR-31, pre-miR-150, or pre-miR-184 reduced ischemia induced retinal or laser induced CNV [54].

miRNA expression profiles were specifically evaluated in the neuroretinal and retinal endothelial cells of the streptozotocin-induced diabetic rat model 3 months after the onset of diabetes, and approximately 100 miRNAs were found to be significantly and differentially expressed in both tissue types. Notably, miRNAs responsive to NF-κB (miR-146, miR-155, miR-132, and miR-21), VEGF (miR-17-5p, miR-18a, miR-20a, miR-21, miR-31, and miR-155), and p53 (miR-34 family) signaling were upregulated, consistent with the pathophysiologic signature of diabetic retinopathy [55]. miR-200b was one of the miRNAs determined to be upregulated in the retinas of streptozotocin-induced diabetic rats. In an independent study, the expression of this miRNA, a negative regulator of VEGF, was interestingly found to be decreased in the retinas of streptozotocin-induced diabetic rats 4 weeks post diabetes induction and also decreased in the retinas of human diabetic retinopathy [56].

miR-137 is a tumor suppressor and a regulator of the transcription factor microphthalmia-associated transcription factor (MITF) in uveal melanocytes. This miRNA lies in the melanoma susceptibility region on chromosome 1, and it is downregulated in uveal melanoma cell lines. Ectopic expression of miR-137 was shown to downregulate MITF, c-Met, and CDK6 and to induce cell cycle arrest. These results suggest that loss of miR-137 activity may be a component of uveal melanoma tumorigenesis [57]. The miR-17/92 cluster is an oncogenic miRNA cluster that is overexpressed in human retinoblastoma and a variety of other human cancers, and overexpression of miR-17/92 in Rb/p107 double knockout mice led to rapid development of retinoblastoma [58]. The studies of miRNA signatures in ocular diseases are summarized in (Table **2**).

## CONCLUSION

Though the complete spectrum of epigenetic modifications remains to be more fully explored, it is clear that epigenetic dysregulation is an important contributor to a variety of common ocular diseases. Many of these diseases, including AMD and diabetic retinopathy, have contributions from environmental risk factors, and alterations in epigenetic patterning may indeed be the mechanistic link between environmental exposures and gene expression changes that set the stage for the development or progression of disease. Potentially disease-relevant DNA methylation, histone modification, and microRNA alterations have been identified. However, a comprehensively characterized epigenomic signature for any ocular disease remains elusive. Although epigenetic therapies have proven particularly effective in cancer applications, [59] the benefits of these approaches have not yet been applied for human ophthalmic diseases. More thorough investigations are needed to expand our collective knowledge of epigenetic modifications in ocular development, homeostasis, and diseases to identify early biomarkers of disease and novel therapeutic targets.

## Figures and Tables

**Table 1.** DNA Methylation and Histone Modifications in Ocular Diseases.

Gene	Modification	Study Population	Tissue	Effect/significance	Reference
*IL17RC*	Hypomethylation of promoter region	AMD patients	Peripheral blood mononuclear cells	Increased frequency of IL-17RC+CD14+ mononuclear cells in peripheral blood	[16]
*GSTM1 *and *GSTM5*	Hypermethylation of promoter region	AMD patients	RPE/choroid and neurosensory retina	Decreased mRNA and protein levels of GSTM1 and GSTM5	[17]
*CRYAA*	Hypermethylation of CpG island at -856 to -640	Age-related cataract patients	Lens epithelial cells	Decreased mRNA and protein levels of CRYAA	[18]
*TGM2*	Hypermethylation of CpG sites at -268, -32, -29 bp	Pterygium patients	Pterygium tissue	Decreased mRNA and protein levels of TGM2	[19]
*MMP2*	Hypomethylation of CpG sites at +484 and +602 bp	Pterygium patients	Pterygium tissue	Increased mRNA and protein levels of MMP2	[19]
*CD24*	Hypomethylation of CpG sites at -809, -762, -631, -629 bp	Pterygium patients	Pterygium tissue	Increased mRNA and protein levels of CD24	[19]
*MSH6*, *CD44*, *PAX5*, *ATA5*, *TP53*, *VHL*, *GSTP1*, *GMT*, *RB1*, and *CDKN2*	Hypermethylation of promoter regions	Retinoblastoma patients	Formalin-fixed paraffin-embedded retinoblastoma tissue	Epigenetic dysregulation of tumor suppressors	[20]
*CXCR4*	Hypermethylation of CpG site in promoter region	Balb/c NOD SCID mice	LS174T human colon adenocarcinoma cells injected into anterior chamber	Ocular microenvironment can regulate promoter methylation and expression of *CXCR4*	[21]
*Sod2*	Increased H4K20me3 and H3K9ac at promoter and enhancer regions	Streptozotocin-induced diabetic rat	Retina	Decreased *Sod2* expression	[22]

**Table 2.** miRNA Signatures in Ocular Diseases.

miRNA	Modification	Study Population	Tissue	Effect/significance	Reference
miR-96, miR-182, miR-183	Expression downregulated	Multiple mouse models of retinitis pigmentosa: rho-/-, Δ307-rds, rds-/-, P347S-Rhodopsin	Retina	These miRNAs are part of a sensory-organ specific cluster and are normally highly expressed in retina	[39, 40]
miR-1, miR-133, miR-142	Expression upregulated	Multiple mouse models of retinitis pigmentosa: rho-/-, Δ307-rds, rds-/-, P347S-Rhodopsin	Retina	Unknown	[39, 40]
let-7	Expression upregulated	Age-related cataract patients	Lens epithelial cells	Increased expression correlated with cataract severity	[41]
miR-142-5p, miR-21	Expression upregulated	Mouse model of experimental autoimmune uveoretinitis	Eye	miR-21 targets IL-12p35	[46]
miR-182	Expression downregulated	Mouse model of experimental autoimmune uveoretinitis	Eye	miR-182 may target IL-17A	[46]
miR-23, miR-27	Locked nucleic acid-modified anti-miRNA (LNA-anti-miR) mediated knockdown	Mouse model of laser-induced choroidal neovascularization	Eye after intravitreal injection of LNA-anti-miRs	Knockdown suppresses development of retinal vasculature, protects against laser-induced choroidal neovascularization	[53]
miR-106a, miR-146, miR-181, miR-199a, miR-214, miR-424, miR-451	Expression upregulated	Mouse model of ischemia-induced retinal neovascularization	Retina	Unknown	[54]
miR-31, miR-150, miR-184	Expression downregulated	Mouse model of ischemia-induced retinal neovascularization	Retina	Intraocular injection of pre-miR-31, 150, or 184 reduces ischemia induced retinal or laser induced CNV	[54]
miR-200b	Expression downregulated	Streptozotocin-induced diabetic rat and human diabetic retinopathy patients	Retina	miR-200b mimic prevents diabetes-induced increase in VEGF	[56]
miR-17/92	Expression upregulated	Human and murine retinoblastoma	Retinoblastoma tissue	Overexpression in Rb/p107 double knockout mouse accelerates retinoblastoma development	[58]
